# Critical Role of COI1-Dependent Jasmonate Pathway in AAL toxin induced PCD in Tomato Revealed by Comparative Proteomics

**DOI:** 10.1038/srep28451

**Published:** 2016-06-21

**Authors:** Min Zhang, Jin Koh, Lihong Liu, Zhiyong Shao, Haoran Liu, Songshen Hu, Ning Zhu, Craig P. Dufresne, Sixue Chen, Qiaomei Wang

**Affiliations:** 1 Key Laboratory of Horticultural Plant Growth, Development and Quality Improvement, Ministry of Agriculture, Department of Horticulture, Zhejiang University, Hangzhou 310058, China; 2 Proteomics and Mass Spectrometry, Interdisciplinary Center for Biotechnology Research, University of Florida, Gainesville, FL 32610, USA; 3 Department of Biology, Genetics Institute, University of Florida, Gainesville, FL 32610, USA; 4 Thermo Fisher Scientific, West Palm Beach, Florida 33407, USA; 5 Zhejiang Provincial Key Laboratory of Horticultural Plant Integrative Biology, Department of Horticulture, Zhejiang University, Hangzhou 310058, China

## Abstract

*Alternaria alternata* f.sp. *Lycopersici* (AAL) toxin induces programmed cell death (PCD) in susceptible tomato (*Solanum lycopersicum*) leaves. Jasmonate (JA) promotes AAL toxin induced PCD in a COI1 (coronatine insensitive 1, JA receptor)-dependent manner by enhancement of reactive oxygen species (ROS) production. To further elucidate the underlying mechanisms of this process, we performed a comparative proteomic analysis using tomato *jasmonic acid insensitive1* ( *jai1*), the receptor mutant of JA, and its wild type (WT) after AAL toxin treatment with or without JA treatment. A total of 10367 proteins were identified in tomato leaves using isobaric tags for relative and absolute quantitation (iTRAQ) quantitative proteomics approach. 2670 proteins were determined to be differentially expressed in response to AAL toxin and JA. Comparison between AAL toxin treated *jai1* and its WT revealed the COI1-dependent JA pathway regulated proteins, including pathways related to redox response, ceramide synthesis, JA, ethylene (ET), salicylic acid (SA) and abscisic acid (ABA) signaling. Autophagy, PCD and DNA damage related proteins were also identified. Our data suggest that COI1-dependent JA pathway enhances AAL toxin induced PCD through regulating the redox status of the leaves, other phytohormone pathways and/or important PCD components.

Fungi usually produce toxins to damage plant tissues, which are classified as host-selective or non-specific. Host-selective toxins (HSTs) only infect host plants, but non-specific toxins can infect plants whether they are a host or non-host of fungus producing pathogen^1^. *Alternaria alternata* f.sp. *Lycopersici* (AAL) toxin is a kind of HST, it is the main virulence effector produced by AAL, causing stem canker and leaf necrosis on tomato (*Solanum lycopersicum*) plants of the *asc/asc* genotype^2^,^3^, leading to serious crop loss. The recessive allele (*asc/asc*) is associated with a mutation in the single codominant *Alternaria Stem Canker* (*ASC*) gene, which determines the insensitivity of plants to fungal AAL^4^. AAL toxin is one type of sphinganine analogue mycotoxins (SAMs) that are inhibitors of eukaryotic sphinganine N-acyltransferase (acyl-CoA-dependent ceramide synthase), a key enzyme in the sphingolipid ceramide biosynthetic pathway^5^. The disruption of sphingolipid biosynthesis causes marked elevation of free sphingoid bases such as phytosphingosine and sphinganine^6^, which triggers programmed cell death (PCD) in susceptible plant cells^7^. Among the five series (TA, TB, TC, TD, and TE) of AAL toxin, TA is the most common kind of AAL toxin^8^. As an active and controlled cell death essential for plant defense, PCD functions in diverse biological processes that are related to the complicated network of PCD development^9^. However, the role and regulatory mechanisms of PCD in plants are still poorly understood.

During host-pathogen interactions, jasmonate (JA) and ethylene (ET) are associated with defense against necrotrophic pathogens, while salicylic acid (SA) plays a major role in activation of defense against biotrophic pathogens^10^. Several studies showed that JA and ET are closely related to AAL toxin induced PCD^11^. During AAL toxin induced PCD process in *Arabidopsis loh2* mutant (a T-DNA knockout mutant of a homologue of the tomato *Asc* gene), ET-responsive genes were up-regulated within seven hours, but JA pathway related genes were unchanged, and there was no indication of JA accumulation^12^. Egusa *et al.*^13^ reported that methyl-jasmonic acid (MeJA) has a promotional effect on susceptibility of tomato to AAL, suggesting that pathogen might utilize JA signaling pathway for successful infection. Moreover, our previous study showed that JA and ET promoted AAL toxin induced cell death alone, and the receptor-dependent JA signaling promotes PCD through enhancing ET biosynthesis^11^. *jai1* (*jasmonic acid insensitive1*) contains a mutation in the tomato homologue of *Arabidopsis COI1* and fails to express JA-regulated genes in response to wounding and MeJA^14^. Hence, the system of AAL toxin and its susceptible tomato host is an excellent model for studying PCD in pathogen response pathways, as the PCD process can be evaluated in a system absence of pathogen, which greatly simplifies the analysis. Besides, the *jai1* mutant allows us to conduct an extensive study in the role of JA pathway in AAL toxin induced PCD.

Reactive oxygen species (ROS) are well known as toxic metabolic substance that can initiate PCD in plant^15^. In *Arabidopsis*, the responses of the JA signaling mutants *jar1* and *fad3/7/8* to O_3_ have indicated that JA could be an important factor involved in the ROS-dependent lesion propagation^16^, hence it is quite interesting to investigate the relationship between ROS and JA pathway during AAL toxin induced PCD.

Although large scale transcriptome analysis has deepened our understanding of the molecular basis of toxin induced PCD, proteomics is another powerful tool to further reveal the regulatory and metabolic pathways underlying plant development and response to stresses^17^,^18^. In this study, we investigated whether JA promoted AAL toxin induced PCD in a COI1-dependent way, and ROS acted downstream of JA in this process. In addition, a comparative proteomics analysis was performed on AAL toxin treated *jai1* mutant and its WT. The data revealed that JA pathway promoted AAL toxin induced PCD by regulating the ROS status of the leaves and relevant PCD components and/or through other hormone pathways in a COI1-dependent manner.

## Results

### COI1-dependent jasmonate pathway promotes TA induced cell death

**Figure 1:**
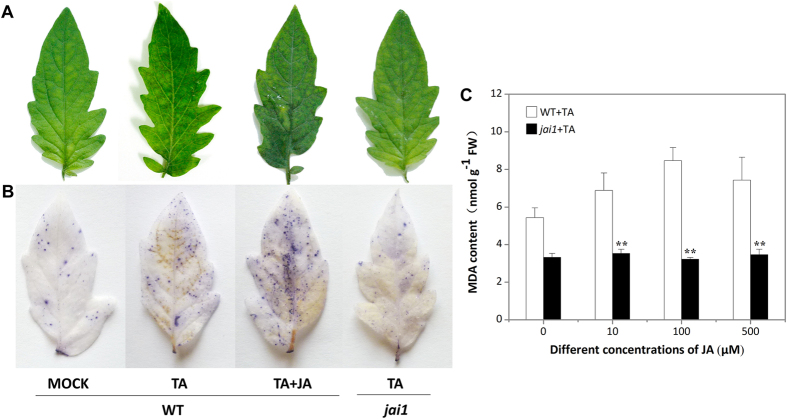
Effect of JA pathway on TA induced PCD in detached tomato leaves. Detached leaflets were incubated under continuous light at 25 °C for 48 h. WT, wild type; *jai1*, JA receptor mutant; Mock, WT leaves treated with SPB buffer; TA, WT or *jai1* leaves treated with TA; TA + JA, WT or *jai1* leaves treated with TA and JA. (**A**) Fully expanded leaflets from nodes between fourth to sixth of 7-week-old plants were treated with 0.2 μM TA with or without 100 μM JA and photographed after 48 h. (**B**) Leaves from WT and *jai1* plants were stained with trypan blue for the degree determination of dead and dying cells after treatment for 48 h. (**C**) MDA content was detected in WT and *jai1* leaflets after co-treated with different concentrations of JA (0, 10, 100, and 500 μM) and 0.2 μM TA for 48 h. Each data point represents the mean of three replicates. Error bars indicate standard deviation of three replicates, asterisks above the bars indicate statistically significant differences between different treatments, as determined by the Student *t*-tests (**P < 0.01).

The visible necrotic lesions were observed at 36 h in leaves of WT plants after TA treatment (data not shown), and the lesions became typical at 48 h. The PCD symptom was enhanced dramatically in WT leaves when treated with TA and JA together in comparison with TA treatment alone (Fig. 1A), suggesting that JA can promote TA induced PCD. Neverthless, TA treated *jai1* leaves displayed minor tissue damage at 48 h, and exogenous JA did not exert effect on it, indicating that COI1 is involved in the PCD process triggered by TA and the impaired JA perception in *jai1* inhibited JA promoted PCD.

Trypan blue staining is an accepted method for cell viability assay, live cells or tissues with intact cell membranes are not coloured, and dead cells can be colored in light blue^19^. As can be seen in Fig. 1B, TA treated WT leaves accumulate more blue precipitate compared with the control, and JA enhanced the accumulation of blue precipitate, indicating that JA can promote TA induced PCD. Besides, TA treated *jai1* leaves accumulated less blue precipitate compared with TA treated WT leaves, which was consistent with the visible phenotype (Fig. 1A), further proved that JA pathway is important for TA induced PCD.

Malondialdehyde (MDA) is the final product of membrane lipid peroxidation, which can be a marker for oxidative stress^20^. MDA content in TA treated WT leaves increased with the increase in JA concentration, and peaked at 100 μM JA. However, no visible change was found in *jai1* leaves with the increase in JA concentration (Fig. 1C), indicating that JA enhanced membrane lipid peroxidation in a COI1-dependent way.

### JA enhances ROS accumulation during TA induced PCD

**Figure 2:**
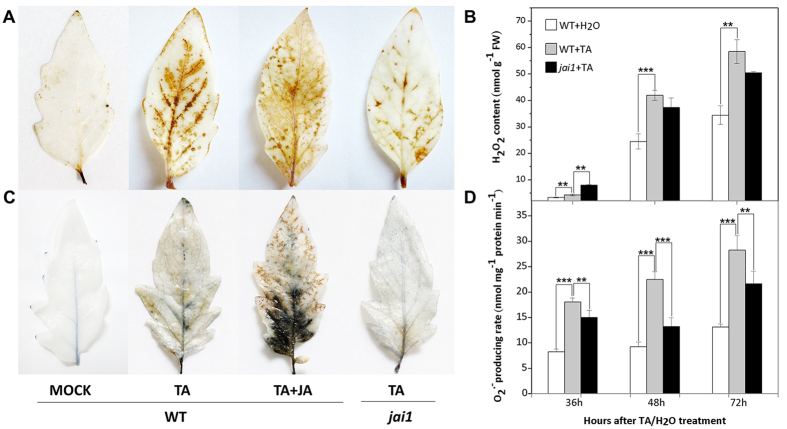
Effect of JA pathway on TA induced ROS accumulation in detached tomato leaves. WT, wild type; *jai1*, JA receptor mutant; Mock, WT leaves treated with SPB buffer; TA, WT or *jai1* leaves treated with TA; TA + JA, or *jai1* leaves treated with TA and JA. (**A**) Leaves of WT and *jai1* were stained with DAB for H_2_O_2_ determination at 48 h after 0.2 μM TA treatment with or without 100 μM JA. (**B**) Changes of H_2_O_2_ content in WT and *jai1* leaves after 0.2 μM TA treatment for 36, 48 and 72 h. (**C**) Leaves of WT and *jai1* were stained with NBT for O_2_^.−^ determination at 48 h after 0.2 μM TA treatment with or without 100 μM JA. (**D**) Changes of O_2_^.−^ producing rate in WT and *jai1* leaves after 0.2 μM TA treatment for 36, 48 and 72 h. Each data point represents the mean of three replicates. Error bars indicate standard deviation of three replicates, asterisks above the bars indicate statistically significant differences between different treatments, as determined by the Student *t-*tests (**P < 0.01, ***P < 0.001).

Hydrogen peroxide (H_2_O_2_) and superoxide (O_2_^.−^) are the two key ROS molecules. We carried out histochemical 3,3′- diaminobenzidine (DAB)^21^ and nitro blue tetrazolium (NBT) staining^22^ to detect H_2_O_2_ and O_2_^.−^ in the leaves, respectively. As shown in Fig. 2A, after TA treatment for 48 h, large brown precipitation was observed to be around the leaf veins of WT plants by DAB staining, and the brown region was expanded by JA treatment. However, *jai1* leaves showed little precipitation after TA treatment, suggesting less ROS production in *jai1*. The H_2_O_2_ content in WT and *jai1* leaves increased steadily during three days with TA treatment, whereas in *jai1* it was significantly lower than WT leaves, except for 36 h after treatment (Fig. 2B). Similarly, much more blue precipitates in TA and JA treated WT leaves was observed than in *jai1* leaves by NBT staining (Fig. 2C). O_2_^.−^ production rate in WT and *jai1* leaves both increased constantly in three days after TA treatment, but it was significantly lower in TA treated *jai1* compared with WT leaves at the same time point (Fig. 2D). Based on these results, it is proposed that ROS burst occurs during TA induced PCD, and the impairment of COI1 suppresses the production of ROS, as well as the necrotic cell death. In Fig. 2B,D, H_2_O_2_ content and O_2_^.−^ production rate in the untreated leaves were also increased slightly, possibly caused by the minor stress brought by the treatment.

### Proteome profiling of tomato leaves

**Figure 3:**
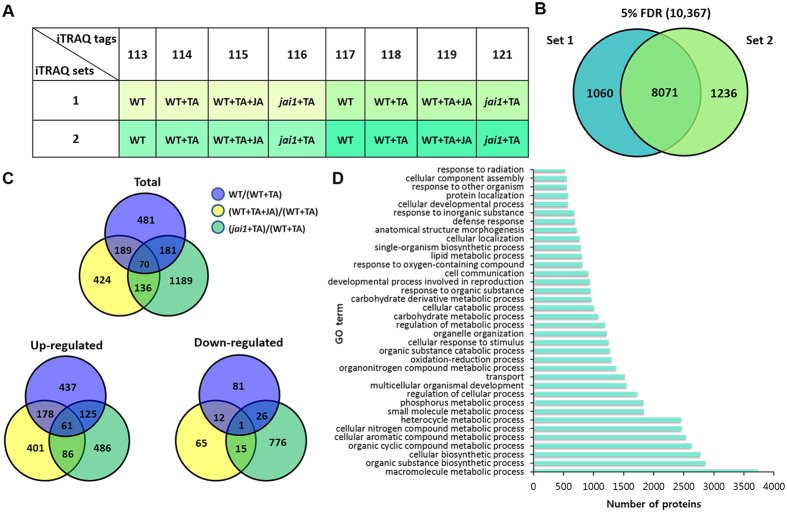
Comparative proteomics identified a large number of proteins involved in TA induced PCD. (**A**) Label by iTRAQ reagent. WT refers to the wild type leaves as control; WT + TA, WT leaves treated with TA; WT + TA + JA, WT leaves treated with TA and JA together; *jai1* + TA, *jai1* leaves treated with TA. Four biological replicates for each treatment and one set of iTRAQ includes two biological replicates. (**B**) Total protein number identified in two sets with 5% false discovery rate (FDR), set 1 is colored in blue, set 2 is colored in green. (**C**) Distribution and overlap of differentially expressed (DE) proteins in each comparison. WT + TA was set as the control group for the other groups. Each color represents one contrast. Blue, WT vs WT + TA; Yellow, WT + TA + JA vs WT + TA; Green, *jai1* + TA vs WT + TA. (**D**) GO terms distribution of the overall 10367 proteins categorized in “biological process”. GO-terms were categorized by Blast2Go at level 4 according to their corresponding GO-term (biological process). Each protein may be identified in more than one process.

To better understand the mechanisms in TA response of tomato leaves and how JA influences the process, a comparative proteomic analysis was performed in JA insensitive mutant *jai1* and its WT after TA treatment with or without JA treatment. We employed high-resolution MS in combination with isobaric tags for relative and absolute quantitation (iTRAQ) proteomics approach to identify the proteome changes. In our workflow, there are four biological replicates for each treatment and one set of iTRAQ includes two biological replicates (Fig. 3A). We identified a total of 8501 tomato proteins with 1% global false discovery rate (FDR) (Supplementary Figure S1) and 10367 tomato proteins with 5% FDR (Fig. 3B), and the 10367 proteins were chosen for the subsequent analysis.

### Functional classification of the 10367 proteins

The identified proteins were analyzed based on the assigned functions of the proteins using Uniprot, NCBI and phytozome databases. The biological process of the overall 10367 proteins were classified into 36 categories sorted by the Blast2Go level 4 (Fig. 3D), among them most proteins were involved in macromolecule metabolic process (36%), organic substance biosynthetic process (28%) and cellular biosynthetic process (27%), regulation of cellular process (17%), oxidation-reduction process (12%) and cellular response to stimulus (12%). Interestingly, there were 9% proteins involved in cell communication, 8% proteins involved in the lipid metabolic process, 7% proteins involved in the defense response, other proteins were involved in transport (15%), organelle organization (12%), developmental process including reproduction (9%) and anatomical structure morphogenesis (7%).

### Subcellular localization of the 10367 proteins

The identified proteins were further classified according to their sub-cellular localization (Supplementary Figure S4). As shown in the graph, the proteins were mainly located in nucleus and chloroplast (both 16%), followed by cytosol, mitochondrion and membrane (above 8%), cytoplasm (7%), plasma membrane (6%), golgi (6%), vacuole (4%), ribosome (3%), cell wall (3%) and so on, while 7% had no specified sub-cellular localization. Significantly, the percentage of proteins located in plasmodesma is 2%, this location may indicated the communication between cells.

### Functional annotation and KEGG pathway analysis of the 10367 proteins

The Kyoto Encyclopedia of Genes and Genomes (KEGG) is a collection of pathways for understanding high-level functions and utilities of the biological system from genomic and molecular-level information^23^. We conducted KEGG pathway analysis of the identified proteins. The proteins were mapped to a total of 139 maps (pathways with more than 40 sequences were represented in Table 1, the others were represented in Supplementary Table S5). The proteins were annotated in various pathways relevant to the synthesis, metabolism or degradation of nucleotide (12%), amino acid (22%), carbohydrate (6%), lipid (14%), secondary metabolites (31%), energy (9%) and xenobiotics (6%). Especially, the pathways of the immune system included the T cell receptor signaling pathway and biotin metabolism pathway. In addition, phosphatidylinositol signaling system and glycosylphosphatidylinositol (GPI)-anchor biosynthesis were also identified in our study.

### Characteristics of the differentially expressed proteins

There were 2670 differencially expressed (DE) proteins (Supplementary Table S4) out of the 10367 proteins (Supplementary Table S3) between the treatment and control samples. The comparison design is displayed as Fig. 3C, WT treated with TA (WT + TA) is the control group for all treated group including WT group. WT vs WT + TA represented proteome changes in WT upon toxin treatment; JA and TA treated WT (WT + TA + JA) vs WT + TA represented JA induced proteome changes upon TA treatment; *jai1* treated with TA ( *jai1* + TA) vs WT + TA showed the COI1-dependent proteome changes during the response of plant to TA. 2400 out of 10367 proteins were excluded from the quantitative analysis because variations were detected within biological replicates within each treatment (Supplementary Figure S2).

Among the DE proteins, there were 921, 819, 1576 proteins in WT vs WT+TA, WT + TA + JA vs WT + TA, and *jai1* + TA vs WT + TA, respectively (Fig. 3C). It indicates that the COI1 mutation can give rise to the largest changes to tomato proteome. Interestingly, most DE proteins in WT after TA treatment or TA + JA treatment showed higher number of increasing patterns, while those in *jai1* after TA treatment exhibited similar number of increasing and decreasing patterns compared with WT. The expression patterns of the differentially expressed proteins in each comparison groups were clustered and displayed as a heat map in Supplementary Figure S5. Among the increased proteins, 61 were shared by the three comparisons, whereas only one protein was found to be decreased in all the comparisons (Fig. 3C), each comparison group also contained their own DE proteins. These proteins present a global view of WT and *jai1* proteome level responses to TA and JA treatment. The expression patterns across each contrasts were classified into 24 categories, as was shown in Supplementary Table S1.

The biological process classification of the 10367 proteins was used as the reference for the functional analysis of DE proteins. We have presented the comparison of functional classification between the 10367 overall detected and 2670 differentially expressed proteins in Supplementary Figure S3, and we found that the biological function processes of the 2670 DE proteins distributed similarly with the overall identified proteins.

### JA-regulated proteins in TA induced PCD process

As shown by the previous physiological results that JA promoted PCD, we further investigated JA-regulated proteins in TA induced PCD process, by checking the DE proteins in WT + TA + JA vs WT + TA comparison. The DE proteins related to cell death, JA, ET, ABA, DNA repair and resistance were listed in Table 2. In animal systems, cysteinyl aspartate-specific proteinases (caspases), serine proteases and specific protease inhibitors play crucial roles during the regulation of PCD process^24^. We found that several proteases were increased by JA treatment, including a kunitz-like protease partial, a cysteine proteinase RD19a-like, an aspartic proteinase nepenthesin-1-like and a β-1, 3-glucanase. Previous study showed that some caspase-like activities are attributable to plant subtilisin-like proteases (SBTs), which are related to cell death^25^. We detected four SBT or SBT like proteins in tomato leaves which were increased by JA treatment. Furthermore, cysteine protease inhibitor (CPI) is known as one of the specific inhibitors of PCD in plant cells^26^. In our experiment, CPI was increased by 23 fold in the WT leaves after treatment with JA and TA together, while increased by two fold after singly TA treatment. These results indicated that TA treatment also induces the expression of some PCD supressors, which could be enhanced by JA treatment. In addition, two JA response proteins, αβ-hydrolases superfamily protein and biotin carboxylase chloroplastic-like, were found to be involved in TA induced PCD. An ET synthesis related protein, probable N-succinyldiaminopimelate aminotransferase-like, was up-regulated by TA + JA treatment compared with TA treatment alone. Four abscisic acid (ABA) response proteins (annexin D4-like, plastid-lipid-associated chloroplastic-like, stem-specific protein TSJT1-like, and PP2A 65 kda regulatory subunit αβ isoform-like) were also increased by JA treatment in response to TA, indicating the possible involvement of ET and ABA pathway in TA induced PCD.

Pathogenesis-related proteins (PRs) are general markers for basal defense response. Two PR10 proteins increased significantly after TA + JA treatment compared with TA treatment alone, while PR-sth2-like protein and PR1 were decreased by JA treatment. Besides, several late blight resistance proteins, a chitinase and a harpin binding protein were also increased by TA + JA treatment, implying that JA could promote the synthesis of resistance proteins. The DNA damage response (DDR) plays an important role against detrimental effects of stress^27^. Three DNA repair related proteins (DNA repair ATPase-related family protein, DNA damage-binding protein 1-like, and DNA mismatch repair protein), were increased by JA during TA induced PCD.

### COI1-dependent signaling pathways in response to TA

We were interested in COI1-dependent signaling pathways in response to TA, as previous results have shown that COI1 impairement inhibits TA induced PCD in tomato leaves. DE proteins between TA treated *jai1* and WT were classified as COI1-dependent proteins in response to TA. The candidate proteins related to defense response and programmed cell death were listed in Table 3. Firstly, six reductase proteins (peroxidase 51-like, glutathione s-transferase T1-like, 2-cys peroxiredoxin, BAS1-chloroplastic-like, probable glutathione s-transferase, mannose- 6-phosphate isomerase 1-like) were significantly higher in *jai1*, whereas an oxidase protein (monooxygenase 1) was decreased in *jai1* compared to WT after TA treatment, suggesting that ROS may act downstream of COI1 to promote TA induced PCD process.

The levels of two ceramide synthesis related proteins, melibiase family protein and serine palmitoyltransferase, were higher in *jai1* than in WT after TA treatment, indicating that COI1-dependent JA signaling might promote TA induced PCD by regulating the biosynthesis of ceramide. Proteins related to autophagy, which plays a role in maintaining the intracellular homeostasis, were also found among the list of DE proteins. Autophagy-related protein 11 (ATG 11) was decreased in *jai1*, whereas RIBONUCLEASE 2-LIKE as a negative regulator of autophagy, was increased in *jai1* after TA treatment. The results indicated that autophagy might be promoted by COI1-dependent JA signaling, and related to inhibition of TA induced PCD. Kunitz Trypsin Inhibitor (KTI1) was previously proved to be an antagonist of cell death triggered by phytopathogens and Fumonisin B1 in *Arabidopsis*^28^. In the current survey, it was increased in *jai1* by two folds. Metacaspase type II and the peptidase C14 caspase catalytic subunit P20, as potential PCD regulators, were increased slightly in *jai1*. This result was different from the former report that the type-II metacaspase from tomato (LeMCA1) was not increased during chemical induced PCD in suspension-cultured tomato cells^29^. As tomato genome contains at least two type-II metacaspases, we speculate that different metacaspases might have distinct functions during PCD process, and display different expression patterns. Most of the above results partly explained why PCD was lighter in *jai1* than in WT.

Our previous studies have shown that JA, ET and SA are involved in the response of tomato leaves to AAL or AAL toxin^30^. In the current survey, we paid more attention to the proteins relevant to these three hormone pathways. The expression of two JA biosynthetic genes, 12-oxophytodienoate reductase 3 and allene oxide synthase were increased in *jai1*, while some of the JA response genes were decreased, due to the impaired perception of JA. ET biosynthesis and response were inhibited, whereas SA synthesis and response were enhanced in *jai1* mutant, suggesting that COI1-dependent JA signaling acts synergistically with ET, and antagonistically with SA during response to TA. Several proteins in ABA response pathway, were decreased in *jai1* after TA treatment (Table 3), indicating that COI1-dependent JA signaling might interact with ABA pathway in regulating TA induced PCD.

## Discussion

Previous studies have shown that AAL toxin can inhibit ceramide biosynthetic enzymes and lead to PCD in sensitive *asc/asc* tomato species, due to the reduced sphingolipids and accumulated dihydrosphingosine (DHS) and 3-ketodihydrosphingosine (3-KDHS)^31^,^32^. However, the underlying mechanism from toxin perception to PCD process is poorly understood in plants. Our research explored the potential pathways and regulators in AAL toxin induced PCD and the role of COI1-dependent JA signaling pathway in regulating the PCD process.

Proteases specifically the classical proteolytic enzymes called caspases, were reported to be participated in the regulation of animal PCD, implies that proteases may be involved in regulation of plant PCD^33^. Several reports also have proved the involvement of proteases in regulating plant PCD. Both protease activity and cell death were inhibited by soybean trypsin inhibitor, while exogenous application of another serine protease prematurely triggered cell death^34^. In tobacco, inhibition of the induced cysteine protease activity by ectopic expression of a cysteine protease inhibitor (CPI) gene, blocked the PCD triggered either by an avirulent pathogen or by ROS^26^. We identified several JA treatment induced proteases during AAL toxin induced PCD, including a kunitz-like protease, a cysteine proteinase RD19a-like, an aspartic proteinase nepenthesin-1-like. a β-1, 3-glucanase and four SBT or SBT like proteins (Table 2). Moreover, we also found that CPI was increased by two fold after toxin treatment alone, while it was increased by 23 fold after TA and JA treatment. The results suggest that TA treatment promoted the expression of some specific proteases to induce PCD, and JA treatment enhanced the response. However, two other caspase-like proteases, metacaspase type II and the peptidase C14 caspase catalytic subunit P20, were increased slightly in *jai1* and decreased in WT after TA treatment, suggesting that these two proteases may function as negative regulators of plant PCD. Kunitz trypsin inhibitor gene (KTI1) was proved to play a regulatory role in PCD antagonizing pathogen and Fumonisin B1 induced cell death^28^. The KTI1 found in our study was increased in *jai1* by two folds in comparison with WT after TA treatment (Table 3), implying that KTI1 is also a negative regulator of plant PCD induced by TA.

Mitochondrial quality control is important in maintaining proper cellular homeostasis, and selective mitochondrial degradation by autophagy (mitophagy) is suggested to play an important role in quality control^34^. Selective autophagy includes the cytoplasm to vacuole targeting (Cvt) pathway^35^ and pexophagy^36^. To date, 31 autophagy-related genes have been identified, which function as the molecular machinery for autophagy. Among them, ATG 11 is essential for mitophagy, acting as an adaptor protein that is needed along with ATG 19 to recruit the Cvt complex to phagophore assembly site (PAS), where the sequestering cytosolic vesicles are generated^37^. In the present study, ATG 11 in tomato was was found to be decreased by COI1 impairment after AAL toxin treatment. Conversely, the autophagy negative regulator RIBONUCLEASE 2-LIKE was significantly increased by COI1 impairment during AAL toxin induced PCD, suggesting that autophagy in *jai1* was suppressed, leading to reduced cell death.

In addition to the putative PCD regulators conserved throughout animal and plant, there also exist some plant-specific mediators of PCD. Various plant hormones are strong candidates, and supporting evidence began to accumulate^38^. Our previous studies showed that COI1-dependent JA pathway acts upstream of ET to promote TA-triggered PCD^11^, which was further verified by the proteomic evidence in this study. The modulator of AAL cell death 1 (MACD1), which is an AP2/ERF transcription factor acting downstream of ET signaling, has been reported to positively regulate AAL triggered cell death^29^. We found that an AP2/ERF transcription factor RAP2-7-like was decreased significantly in *jai1* during response to TA, suggesting that COI1-mediated JA signaling promote TA induced PCD by enhancement of ET response via RAP2-7 like. The resistance of tomato plants to AAL and AAL toxin is enhanced by SA pathway^30^. The proteins related to SA biosynthesis and response were increased in *jai1* mutant after TA treatment, indicating that COI1-dependent JA pathway interacts with SA pathway in an antagonistic way to enhance TA induced PCD.

ROS have emerged as important signals in activation of plant PCD. Studies on exogenous application of H_2_O_2_ confirmed the role of H_2_O_2_ as a cell death trigger^39^. Zhang^40^ also proved that AAL toxin induced PCD is closely related to the production of ROS. In the present study, the decreased levels of H_2_O_2_ and O_2_^.−^ in *jai1* is consistent with the reduced cell death in *jai1* after AAL toxin treatment, suggesting that JA exert its effects on plant PCD through regulation of ROS accumulation. Inhibitors of ET biosynthesis or perception blocked H_2_O_2_ production and cell death in tomato suspension cells^41^, therefore the decreased ROS production in *jai1* might be owing to the inhibited ET pathway. In addition, JA also acted through regulation of ET pathway in many cases, for example, JA-promoted lycopene was correlated with JA-stimulated ET production^42^, and both ET biosynthesis and the signaling pathway are strongly decreased in *jai1* leaves inoculated with AAL when compared with WT^30^. The *Arabidopsis* jasmonate-insensitive mutant *jar1* shows enhanced cell death after exposure to O^3−^, and wounding or treatment with JA has been shown to reduce O^3−^ induced cell death and ROS levels^16^, in contrast to our finding in TA induced PCD. Complex mechanisms exist among PCD processes induced by distinct factors, which may be species-specific. Besides JA and ET, ABA seems to act downstream of COI1-mediated JA signaling in promoting TA induced PCD. Virus induced gene silencing (VIGS) analyses proved that AAL toxin triggered cell death is dependent on the mitogen-activated protein kinase MEK2 in tobacco^43^. We observed that the mitogen-activated protein kinase 9-like (MAPK9-Like) was significantly decreased in *jai1* after TA treatment, suggesting a possible role of MAPK9-Like in response to TA in tomato leaves.

The CCR4-NOT transcription complex has been well known as mRNA deadenylases in eukaryotic cells. Liang *et al.*^44^ proved that the homologs of CCR4-associated factor 1 (CAF1) in Arabidopsis are involved in defence responses to pathogen infections. In our results, two subunits of CCR4-NOT transcription complex were upregulated in *jai1* mutant compared with the wild type (Supplementary Table S4), indicating that the CCR4-NOT transcription complex is also involved in the defence responses to AAL toxin in tomato.

In plants, plasma membrane (PM) H^+^-ATPases are the primary pumps responsible for the establishment of cellular membrane potential, which are absolutely essential for normal plant growth and development^45^. A PM H^+^-ATPase was slightly decreased by TA treatment, suggesting its function in toxin response. Moreover, if DNA damage is left unrepaired or mis-repaired, it can be changed into a mutation. Three DNA damage response related proteins were found to be increased by JA, indicating that JA promotes the DNA repair process to cope with the occurrence of the PCD and mutation.

**Figure 4:**
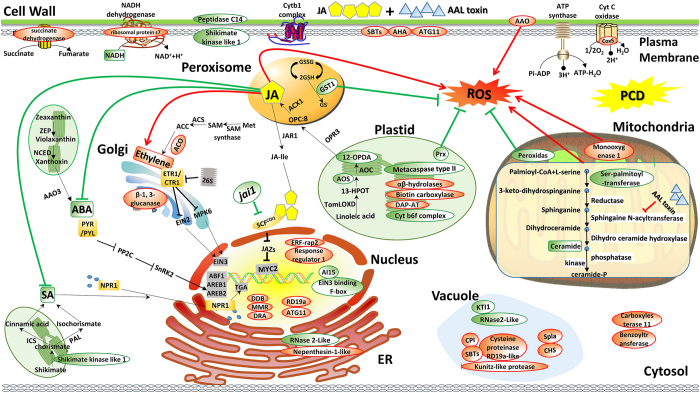
A putative model showing possible mechanisms of JA regulated AAL toxin response in *Solanum Lycopersicum*. The DE proteins caused by JA treatment and COI1 mutation during AAL toxin induced PCD were used to construct the model, and the impairment of COI1 induced protein changes were presented with the contrary change patterns. The identified proteins were assigned to different organelles or groups according to their subcellular localization and molecular functions. Up-regulated proteins are highlighted red and down-regulated proteins are colored green. Arrows and bars represent positive and negative regulation respectively. Red solid lines indicate that the pathways are up-regulated or promoted by JA during TA response, green solid lines with bars or arrows indicate that the pathways are down-regulated by JA during TA response, black solid lines with arrows links the proteins within the same pathway. TA induce PCD by inhibiting the synthesis of ceramide and inducing the overproduction of ROS, COI1-dependent JA pathway may promote this PCD progress by influencing the ROS production and scavenging, other hormone signaling pathways or some possible PCD regulators such as caspase-like proteins, autophagy and DNA repair related proteins. AAO, L-ascorbic acid oxidase; ABI1, ABA insensitive1; ACO1, 1-aminocyclopropane-1-carboxylate oxidase homolog 1-like; AHA, plasma membrane H^+^-ATPase; AOS, allene oxide synthase; ATG11, autophagy related protein 11; CHS, chalcone synthase; CPI, cysteine protease inhibitor; Cyt, Cytochrome; DAP-AT, probable n-succinyldiaminopimelate aminotransferase-like; DDB, DNA damage binding proteins; DDR, DNA damage response; DRA, DNA repair ATPase-related family protein; ER, endoplasmic reticulum; GST, glutathione S-transferase; Golgi, golgi complex; KTI1, kunitz trypsin inhibitor; MMR, DNA mismatch repair; NAT, nucleobase-ascorbate transporter 6-like; Prx, Peroxiredoxin; RNase E, Ribonuclase 2-Like; SBTs, subtilisin-like proteases; TF, transcription factor.

In summary, we explored the mechanisms in JA regulation of AAL toxin induced PCD using comparative proteomics. We identified a large number of DE proteins induced by TA + JA treatment in WT and *jai1*. The DE proteins revealed by iTRAQ quantitative proteomics approach in this study help to elucidate the molecular regulating network of COI1-dependent JA pathway in PCD. Numerous new components in cell death machinery were identified, which were summarized in several pathways and represented in Fig. 4. TA causes PCD by inhibiting the synthesis of ceramide and inducing the overproduction of ROS. COI1-dependent JA pathway may promote this PCD progress by influencing the ROS production and scavenging, other hormone signaling pathways or some possible PCD regulators such as caspase-like proteins, autophagy and DNA repair related proteins. Our findings have deepened the understanding of the mechanisms in fungal toxin induced PCD and JA mediated plant defense in response to the fungal toxin. Many of the proteins identified in the present study including their modifications are interesting targets for further genetic and molecular studies to establish the precise roles in cell death and defense regulatory networks.

## Methods

### Plant growth and selection of *jai1* homozygotes

Tomato (*S. lycopersicum*) cultivar Castlemart (CA) is the parental line for JA insensitive mutant *jai1*. Seeds were germinated on the filter paper after treatment with 1% sodium hypochlorite (NaOCl) for 10 minutes. The germinated seedlings were treated with 1 mM MeJA (Sigma, St Louis, MO, USA). Approximately 24 h or 36 h later, MeJA-insensitive seedlings (roots growth is not inhibited by MeJA) were selected by PCR using genomic DNA using three primers below: P1: 5′-GTGGAGACGATATGTTGAGACTAA-3′; P2: 5′-CCATGGAG TCCATCACCTAACAGT-3′; P3: 5′-GTGGTCAGATCAGAGCCCTCTATT-3′; PCR product with only a 777 bp band are homozygous *jai1* mutant. Seedlings were grown in the growth chamber with day/night temperature of 26/18 °C (16/8h). All experiments were carried out using fully expanded leaflets from nodes 4–6 (except for the terminal leaflets) of 7-week-old tomato plants. *jai1* homozygotes were screened according to Li *et al.*^14^.

### Detached leaflet treatment with JA and AAL Toxin

Treatment was performed as described earlier^46^,^47^. The treatment solutions contained different concentrations of JA (0, 10, 100, or 500 μM) and 0.2 μM TA (the most common kind of AAL toxin^8^) under continuous light at 25 °C, sodium phosphate buffer (SPB, pH 7.0) was used as a control. Four excised leaflets from individual plants were incubated for different time periods on a piece of filter paper in one Petri dish.

### Cell death assays

Cell death was detected using trypan blue staining. Detached leaves were submerged in the farmers solution (acetic acid:ethanol:chloroform = 1:6:3, V/V/V) to make it transparent, dyed in the solution involving 0.05% w/v trypan blue and ethanol (1:2 V/V), and then washed with water and decolorized in saturated chloral hydrate.

### Detection of malondialdehyde content

Leaf samples (0.4 g each) were ground in 4 mL of phosphate buffer (0.05 M PBS, pH 7.8, 0.2 mM EDTA, 2% polyvinyl pyrrolidone), centrifuged at 12000 *g* for 20 min and the total supernatant was used for the measurement. The measurement of malondialdehyde (MDA) content was calculated from the thiobarbituricacid (TBA) reaction using an extinction coefficient of 155 mM^−1^ cm^−1 ^48. One milliliter supernatant was added into 3 mL of TBA solution, kept at 95 °C for 30 min, centrifuged at 1500 *g* for 10 min, and then MDA content was calculated according to the absorbance measured at 600 nm, 532 nm, and 450 nm, respectively.

### Detection and quantification of H_2_O_2_ and superoxide

Intracellular H_2_O_2_ was detected by 3, 3′-diaminobenzidine tetrahydrochloride (DAB) staining, showing a brown stain caused by the polymerization of DAB^21^. Superoxide (O_2_^.−^) was detected by staining leaves with nitro bluetetrazolium chloride (NBT), which is reduced by superoxide to form a dark blue, water-insoluble formazan^22^. The O_2_^.−^ production rate was measured by analyzing the nitrite formation from hydroxylamine in the presence of O_2_^.−^. Frozen leaf segment was homogenized with 3 mL of 65 mM Phosphate Buffered Saline (PBS, pH 7.8) and centrifuged at 5000 *g* for 10 min. After incubation in the solution containing 0.9 mL of 65 mM PBS (pH 7.8), 0.1 mL of 10 mM hydroxylamine hydrochloride, and 1 mL of the supernatant at 25 °C for 20 min, 17 mM sulphanilamide and 7 mM anaphthylamine were added. Ethylether in the same volume was added and centrifuged at 1500 *g* for 5 min. The absorbance in the aqueous solution was monitored at 530 nm^49^. Quantification of H_2_O_2_ was done as follows: 0.4 g samples were ground in cold acetone and centrifuged at 3000 *g* for 10 min, the supernatant was then mixed with 0.2 mL of 20% TiCl_4_ and 0.4 mL of ammoni, the precipitation was washed with acetone for five times, finally dissolved in 3 mL of 2 M H_2_SO_4_, and determined spectra-photometrically by measuring the absorbance at 595 nm.

### Protein extraction and quantification

Proteins from tomato leaves of four biological replicates were prepared according to Hurkman and Tanaka^50^ with the following modifications. Samples were ground in liquid nitrogen into fine powder and incubated in extraction buffer (0.1 M Tris-HCl pH 8.8, 10 mM EDTA, 0.2 M DL-Dithiothreitol, 0.9 M sucrose), continued by grinding in a fume hood, and then the extract was agitated for 2hrs at room temp. After washing twice with 0.1 M ammonium acetate in methanol and twice with 80% acetone, the dried pellet was dissolved with 2D buffer (8 M Urea, 4% CHAPS, 40 mM Tris-base, 2 M Thiourea). Protein assays were performed using an EZQ Protein Quantitation Kit (Invitrogen, Carlsbad, CA, USA) with the SoftMax Pro Software v5.3 (Molecular Devices, Downingtown, PA, USA).

### Protein precipitation, iTRAQ labeling, strong cation exchange, LC-MS/MS

For each sample, 50 μg protein was dissolved in the dissolution buffer with 1 μl of denaturant in the iTRAQ reagents 8-plex kit (AB Sciex, Inc., Foster City, CA, USA). The samples were reduced with Tris (2-Carboxyethyl) Phosphine (TCEP), alkylated with methyl methanethiosulfonate (MMTS), trypsin-digested, and labeled according to the manufacturer’s instructions for the iTRAQ reagents 8-plex kit (AB Sciex Inc., California, USA). Mock lines (wild type, CA) were labeled with iTRAQ tags 113 and 117; TA treated WT lines were labeled with tags 114 and 118; TA and jasmonic acid treated WT lines were labeled with tags 115 and 119; TA treated *jai1* lines were labeled with tags 116 and 121. The biological quadruplicates were analyzed to account for variation among individuals. The combined peptide mixtures were desalted and lyophilized. After labeling, samples were combined, desalted with solid phase extration (The Nest Group, Inc., Southborough, MA, USA), lyophilized and dissolved in strong cation exchange (SCX) solvent A (25% (v/v) acetonitrile, 10 mM ammonium formate and 0.1% (v/v) formic acid (pH 2.8)). The peptides were fractionated using an Agilent HPLC system 1260 with a polysulfoethyl column (2.1 mm × 100 mm, 5 μL, 300 Å; PolyLC, Columbia, MD, USA), flow rate of 0.2 mL/min. Peptides were eluted with a linear gradient of 0–20% solvent B (25% (v/v) acetonitrile and 500 mM ammonium formate (pH 6.8)) over 50 min, followed by ramping up to 100% solvent B in 5 min. The absorbance at 280 nm was monitored; 12 fractions were collected and lyophilized. The fractions were resuspended in LC solvent A (0.1% formic acid in 97% water, 3% acetonitrile). A hybrid quadrupole Orbitrap (Q Exactive) MS system (Thermo Fisher Scientific, Bremen, Germany) was used with high energy collisional dissociation (HCD) after each MS. The instrument was run in data dependent mode with a full MS (400–2000 m/z) resolution of 70,000 and five ms/ms (17500 resolution, HCD NCE = 28%, isolation width = 3 Th, first mass = 105 Th., 5% underfill ratio, peptide match set to ‘preferred’, and an AGC target of 1e^6^). Dynamic exclusion of 10 s was applied to prevent repeated analyses of the same peptides, and a lock mass of m/z 445.12003 (polysiloxane ion) was used for real-time internal calibration.). The MS system was interfaced with an automated Easy-nLC 1000 system (Thermo Fisher Scientific, Germerling, Germany). Each sample fraction was loaded on an Acclaim Pepmap 100 pre-column (20 cm × 75 μm; 3 μm-C18) and separated using a PepMap RSLC analytical column (250 cm × 75 μm; 2 μm-C18) with a flow rate at 350 nl/min. A linear gradient of solvent A (0.1% formic acid) to solvent B (0.1% formic acid, 99.9% acetonitrile) was run for 95 min, followed by a ramp to 98% B for 5 min. The MS proteomics data have been deposited in the ProteomeXchange Consortium^51^ via the PRIDE partner repository with the data set identifier PXD002864 and 10.6019/PXD002864.

### iTRAQ LC-MS/MS data analysis

The raw MS/MS data files were searched against the specified non-redundant database (combined Uniprot, http://www.uniprot.org/uniprot/?query =  solanum+lycopersicum&sort =  score; NCBI, http://www.ncbi.nlm.nih.gov/gquery/?term=solanum+lycopersicum; Phytozome, http://www.phytozome.net/tomato.php) using the Fraglet and Taglet searches under the Paragon^TM^ algorithm^52^ of ProteinPilot v.4.5 software (AB Sciex, Inc.). Plant species, fixed modification of methylmethane thiosulfate-labeled cysteine, fixed iTRAQ modification of amine groups in the N-terminus and lysine, and variable iTRAQ modifications of tyrosine were considered. In addition, the iTRAQ data were analyzed using Proteome Discoverer v1.4 (Thermo Fisher Scientific, Bremen, Germany) and the following parameters: peptide tolerance at 10 ppm, tandem MS tolerance at ±0.01 Da, peptide charges of 2+ to 5+, trypsin as the enzyme, allowing one missed cleavage, iTRAQ label and methyl methanethiosulfonate (C) as fixed modifications, and oxidation (M) and phosphorylation (S, T, Y) as variable modifications. Peptides and proteins were filtered using ProteoIQ 2.7 (Premier Biosoft, Palo Alto, CA, USA) with strict peptide and protein probabilities, 0.8 and 0.95, respectively. For peptide confidence, we adopted the following cutoff values of Xcorr that are commonly used for the SEQUEST algorithm^53^: 2.31 for 2+, 2.41 for 3+, and 2.6 for 4+ peptides^54^. Peptide probability was applied to filter peptide assignments obtained from MS/MS database searching results using predictable false identification error rate (See Supplementary Table S2 for peptide information). To be differentially expressed with significance, a protein must have been quantified with a fold change >1.2 or <0.8, with at least three spectra in at least two of the biological quadruplicates, along with a Fisher’s combined probability <0.05^55^. A protein is considered to be variable if it has an increase in expression in one component of the comparison and a decrease in the other; these variable proteins within biological replicates were excluded for further analysis. Functional analyses of proteins in biological process, molecular function, and cellular component were conducted using GO annotation (http://www.geneontology.org/) with Fisher’s exact test based on false discovery rate (*p*-value ≤0.05)^56^. Blast2GO level 2–11 filtering was used to examine unique protein changes during comparison analysis. Accession numbers were compared between each genotype and treatment, and unique proteins were identified.

### Statistical analysis

Differences in the MDA content, H_2_O_2_ content and O_2_^.−^ production rate were analyzed by one-way analysis of variance (ANOVA); if the ANOVA analysis was significant (P < 0.05), Duncan’s multiple range test was used to detect significant differences between groups.

## Additional Information

**How to cite this article**: Zhang, M. *et al.* Critical Role of COI1-Dependent Jasmonate Pathway in AAL toxin induced PCD in Tomato Revealed by Comparative Proteomics. *Sci. Rep.* **6**, 28451; doi: 10.1038/srep28451 (2016).

## Supplementary Material

Supplementary Information

Supplementary Table S1

Supplementary Table S2

Supplementary Table S3

Supplementary Table S4

Supplementary Table S5

Supplementary Table S6

Supplementary Table S7

Supplementary Table S8

Supplementary Table S9

## Figures and Tables

**Table 1:** The most enriched Kyoto Encyclopaedia of Genes and Genomes (KEGG) pathways.

Classification^*a*^	KEGG pathways	Sequence number^*b*^
Nucleotide	Purine metabolism	262
	Pyrimidine metabolism	93
	Amino sugar and nucleotide sugar metabolism	93
	Pentose and glucuronate interconversions	74
	Pentose phosphate pathway	68
	Phenylalanine metabolism	147
Amino acid	Glutathione metabolism	87
	Arginine and proline metabolism	77
	Glycine, serine and threonine metabolism	77
	Cysteine and methionine metabolism	71
	Tryptophan metabolism	68
	Valine, leucine and isoleucine degradation	63
	Aminobenzoate degradation	58
	Aminoacyl-tRNA biosynthesis	54
	Tyrosine metabolism	52
	Alanine, aspartate and glutamate metabolism	51
	Lysine degradation	46
	Cyanoamino acid metabolism	46
	β-Alanine metabolism	44
	Phenylalanine, tyrosine and tryptophan biosynthesis	40
Carbohydrate	Starch and sucrose metabolism	212
	Galactose metabolism	82
Lipid	Glycerolipid metabolism	85
	Phosphatidylinositol signaling system	70
	Fatty acid degradation	68
	Inositol phosphate metabolism	59
	α-Linoleic acid metabolism	54
	Glycerophospholipid metabolism	48
	Fatty acid biosynthesis	42
	Biosynthesis of unsaturated fatty acids	41
Secondary metabolites	Pyruvate metabolism	93
	Thiamine metabolism	80
	Glyoxylate and dicarboxylate metabolism	73
	Methane metabolism	71
	Flavonoid biosynthesis	49
	Porphyrin and chlorophyll metabolism	45
	Ascorbate and aldarate metabolism	43
Energy	Glycolysis/Gluconeogenesis	118
	Carbon fixation in photosynthetic organisms	87
	Fructose and mannose metabolism	64
	Oxidative phosphorylation	58
	Carbon fixation pathways in prokaryotes	57
	Citrate cycle (TCA cycle)	48
Xenobiotics	Drug metabolism - cytochrome P450	70
	T cell receptor signaling pathway	66
	Metabolism of xenobiotics by cytochrome P450	65
	Drug metabolism - other enzymes	45

^*a*^KEGG pathways were classified by metabolic pathways of different molecules.

^*b*^Sequence number involved in the corresponding KEGG pathways above 40 were presented.

**Table 2:**
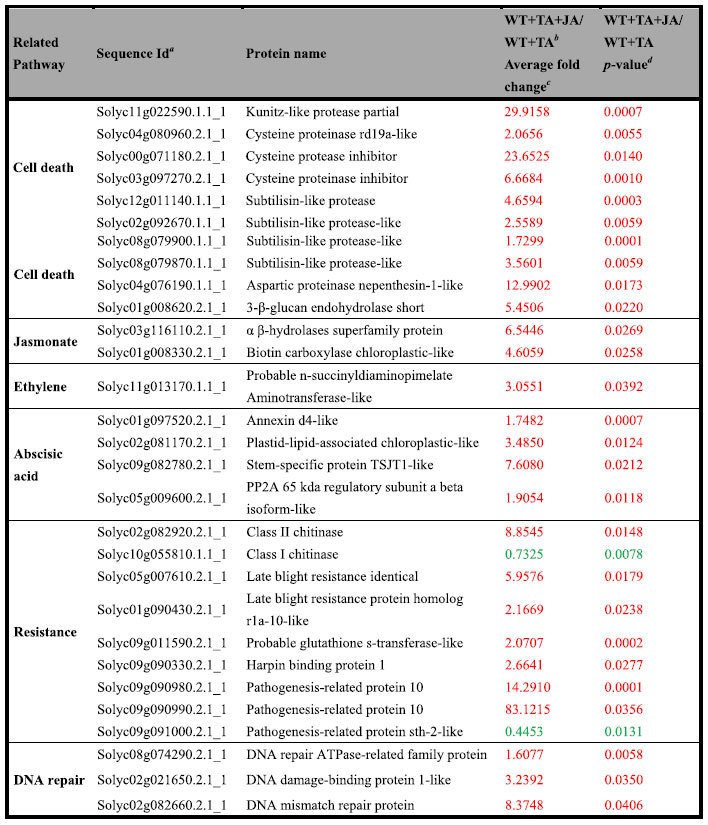
Interesting and novel proteins changed by JA treatment in the process of TA induced PCD.

^*a*^Accession numbers from GenBank database or Sol Genomics Network (SGN).

^*b*^(WT + TA + JA/WT + TA) represented JA and TA treated WT vs TA treated WT.

^*c*^Red color represents that the protein was up-regulated, green color represents that the protein was down-regulated.

^*d*^*t*-test indicates significant difference in expression of these proteins between WT treated with JA and TA and WT treated with TA alone, with p-value threshold values (α = 0.05).

**Table 3:**
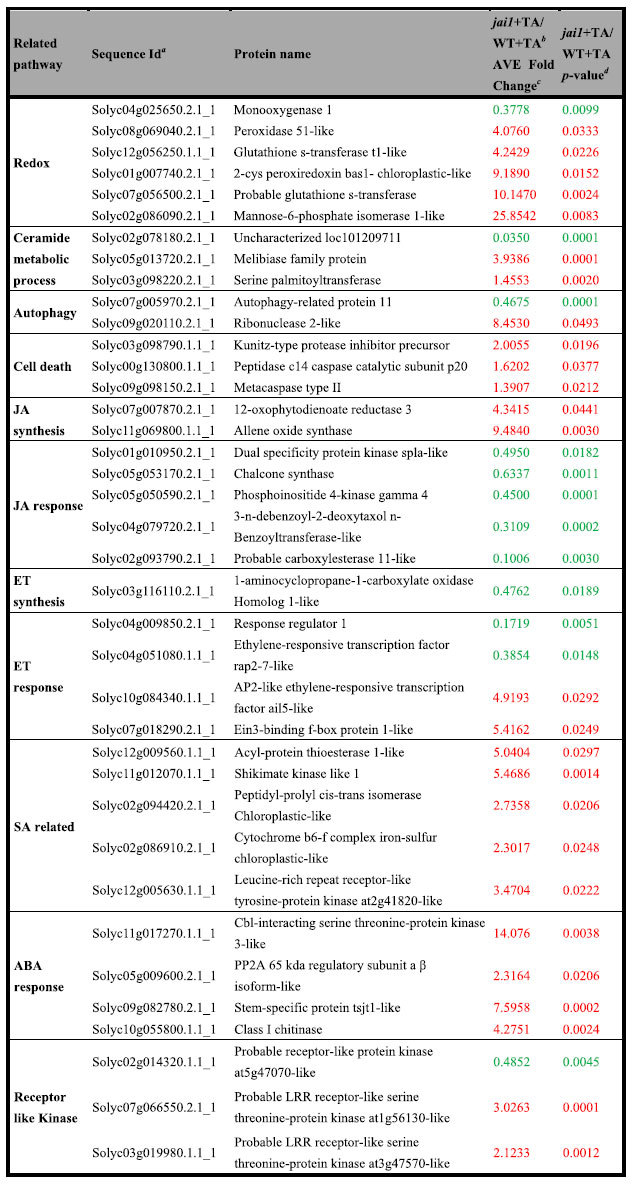
COI1-dependent pathways in response to TA.

^*a*^Accession numbers from GenBank database or Sol Genomics Network (SGN).

^*b*^( *jai1* + TA/WT + TA) represented *jai1* treated with TA vs WT treated with TA.

^*c*^Red color represents that the protein was up-regulated, green color represents that the protein was down-regulated.

^*d*^*t-*test indicates significant difference in expression of these proteins between *jai1* treated with TA and WT treated with TA, with p-value threshold values (α = 0.05).

## References

[b1] Tsuge T. *et al.* Host-selective toxins produced by the plant pathogenic fungus *Alternaria alternata*. FEMS Microbiol. Rev. 37, 44–66 (2013). 22846083 10.1111/j.1574-6976.2012.00350.x

[b2] Brandwagt B. F., Kneppers T. J., Nijkamp H. J. & Hille J. Overexpression of the tomato *Asc-1* gene mediates high insensitivity to AAL toxins and fumonisin B1 in tomato hairy roots and confers resistance to *Alternaria alternata* f. sp. *lycopersici* in *Nicotiana umbratica* plants. Mol Plant Microbe Interact. 15, 35–42 (2002). 11858172 10.1094/MPMI.2002.15.1.35

[b3] Grogan R., Kimble K. & Misaghi I. A stem canker disease of tomato caused by *Alternaria alternata* f. sp. *lycopersici* [Fungus diseases]. Phytopathol. 65 (1975).

[b4] Clouse S. D. & Gilchrist D. G. Interaction of the *asc* locus in F8 paired lines of tomato with *Alternaria alternata* f. sp. *lycopersici* and AAL-toxin. Phytopathol. 77, 80–82 (1987).

[b5] Du L. *et al.* Biosynthesis of sphinganine-analog mycotoxins. J Ind Microbiol. 35, 455–464 (2008). 10.1007/s10295-008-0316-y 18214562

[b6] Abbas H. K. *et al.* Fumonisin-and AAL-toxin-induced disruption of sphingolipid metabolism with accumulation of free sphingoid bases. Plant Physiol. 106, 1085–1093 (1994). 12232389 10.1104/pp.106.3.1085 PMC159634

[b7] Riley R. T., Norred W. P. & Bacon C. W. Fungal toxins in foods: recent concerns. Annu. Rev. Nutr. 13, 167–189 (1993). 8369143 10.1146/annurev.nu.13.070193.001123

[b8] Xu L. & Du L. Direct detection and quantification of *Alternaria alternata lycopersici* toxins using high-performance liquid chromatography-evaporative light-scattering detection. J Microbiol Methods. 64, 398–405 (2006). 16014317 10.1016/j.mimet.2005.06.004

[b9] Tsuda K., Sato M., Stoddard T., Glazebrook J. & Katagiri F. Network properties of robust immunity in plants. PLoS Genet. 5, 61–82 (2009). 10.1371/journal.pgen.1000772 PMC2782137 20011122

[b10] Glazebrook J. Contrasting mechanisms of defense against biotrophic and necrotrophic pathogens. Annu Rev Phytopathol. 43, 205–227 (2005). 16078883 10.1146/annurev.phyto.43.040204.135923

[b11] Zhang L. *et al.* The involvement of jasmonates and ethylene in *Alternaria alternata* f. sp. *lycopersici* toxin-induced tomato cell death. J. Exp. Bot. 62, 5405–5418 (2011). 21865178 10.1093/jxb/err217 PMC3223041

[b12] Gadjev I. Z., Hille J. & Gechev T. S. An extensive microarray analysis of AAL-toxin-induced cell death in *Arabidopsis thaliana* brings new insights into the complexity of programmed cell death in plants. Cell. Mol. Life Sci. 61, 1185–1197 (2004). 15141304 10.1007/s00018-004-4067-2 PMC11138596

[b13] Egusa M., Ozawa R., Takabayashi J., Otani H. & Kodama M. The jasmonate signaling pathway in tomato regulates susceptibility to a toxin-dependent necrotrophic pathogen. Planta 229, 965–976 (2009). 19148670 10.1007/s00425-009-0890-x

[b14] Li L. *et al.* The tomato homolog of *CORONATINE-INSENSITIVE1* is required for the maternal control of seed maturation, jasmonate-signaled defense responses, and glandular trichome development. Plant cell 16, 126–143 (2004). 14688297 10.1105/tpc.017954 PMC301400

[b15] Dat J. F. *et al.* Changes in hydrogen peroxide homeostasis trigger an active cell death process in tobacco. Plant J. 33, 621–632 (2003). 12609037 10.1046/j.1365-313x.2003.01655.x

[b16] Rao M. V., Lee H., Creelman R. A., Mullet J. E. & Davis K. R. Jasmonic acid signaling modulates ozone-induced hypersensitive cell death. Plant cell 12, 1633–1646 (2000). 11006337 10.1105/tpc.12.9.1633 PMC149075

[b17] Zhang A., Xu T., Zou H. & Pang Q. Comparative proteomic analysis provides insight into cadmium stress responses in *brown algae Sargassum fusiforme*. Aquat Toxicol. 163, 1–15 (2015). 25827747 10.1016/j.aquatox.2015.03.018

[b18] Dai S. J., Pang Q. Y., Tian Y. X., Chen S. X. & Yan X. F. Proteomic Analysis of Arabidopsis Leaves Subjected to Mechanical Wounding. Curr. Proteomics 12, 124–136 (2015).

[b19] Tennant J. R. Evaluation of the trypan blue technique for determination of cell viability. Transplantation 2, 685–694 (1964). 14224649 10.1097/00007890-196411000-00001

[b20] Davey M. W., Stals E., Panis B., Keulemans J. & Swennen R. L. High-throughput determination of malondialdehyde in plant tissues. Anal Biochem 347, 201–207 (2005). 16289006 10.1016/j.ab.2005.09.041

[b21] Torres M. A., Dangl J. L. & Jones J. D. Arabidopsis gp91phox homologues AtrbohD and AtrbohF are required for accumulation of reactive oxygen intermediates in the plant defense response. PNAS 99, 517–522 (2002). 11756663 10.1073/pnas.012452499 PMC117592

[b22] Shinogi T., Suzuki T., Kurihara T., Narusaka Y. & Park P. Microscopic detection of reactive oxygen species generation in the compatible and incompatible interactions of *Alternaria alternata Japanese pear* pathotype and host plants. J Gen Plant Pathol. 69, 7–16 (2003).

[b23] Ogata H. *et al.* KEGG: Kyoto Encyclopedia of Genes and Genomes. Nucleic Acids Res. 27, 29–34(26) (1999). 9847135 10.1093/nar/27.1.29 PMC148090

[b24] Shi Y. Mechanisms of caspase activation and inhibition during apoptosis. Mol. Cell 12, 459–470 (2002). 11931755 10.1016/s1097-2765(02)00482-3

[b25] Vartapetian A. B., Tuzhikov A. I., Chichkova N. V., Taliansky M. & Wolpert T. J. A plant alternative to animal caspases: subtilisin-like proteases. Cell Death Differ. 18, 1289–1297 (2011). 21546909 10.1038/cdd.2011.49 PMC3172098

[b26] Solomon M., Delledonne M., Menachem E., Levine A. & Belenghi B. The involvement of cysteine proteases and protease inhibitor genes in the regulation of programmed cell death in plants. Plant cell 11, 431–443 (1999). 10072402 10.1105/tpc.11.3.431 PMC144188

[b27] Czarny P., Pawlowska E., Bialkowska-Warzecha J., Kaarniranta K. & Blasiak J. Autophagy in DNA damage response. Int J Mol Sci. 16(2015). 10.3390/ijms16022641 PMC4346856 25625517

[b28] Li J., Brader G. & Palva E. T. Kunitz trypsin inhibitor: an antagonist of cell death triggered by phytopathogens and fumonisin B1 in *Arabidopsis*. Mol. Plant 1, 482–495 (2008). 19825555 10.1093/mp/ssn013

[b29] Mase K. *et al.* Ethylene-responsive AP2/ERF transcription factor MACD1 participates in phytotoxin-triggered programmed cell death. Mol Plant Microbe Interact. 26, 868–879 (2013). 23617414 10.1094/MPMI-10-12-0253-R

[b30] Jia C. *et al.* Multiple phytohormone signalling pathways modulate susceptibility of tomato plants to *Alternaria alternata* f. sp. *lycopersici*. J. Exp. Bot. 64, 637–650 (2013). 23264518 10.1093/jxb/ers360 PMC3542053

[b31] DV. L. Enzymes of sphingolipid metabolism in plants. Methods in Enzymol. 311, 130–149 (2000). 10563318 10.1016/s0076-6879(00)11074-2

[b32] Wang E., Norred W. P., Bacon C. W., Riley R. T. & A H Merrill J. Inhibition of sphingolipid biosynthesis by fumonisins. Implications for diseases associated with Fusarium moniliforme. J Biol Chem. 266, 14486–14490 (1991). 1860857

[b33] Fuentes-Prior P. & Salvesen G. S. The protein structures that shape caspase activity, specificity, activation and inhibition. Biochem. J. 384, 201–232 (2004). 15450003 10.1042/BJ20041142 PMC1134104

[b34] Groover A. & Jones A. M. Tracheary element differentiation uses a novel mechanism coordinating programmed cell death and secondary cell wall synthesis. Plant Physiol. 119, 375–384 (1999). 9952432 10.1104/pp.119.2.375 PMC32113

[b35] Klionsky D. J. & Emr S. D. Autophagy as a regulated pathway of cellular degradation. Science 290, 1717–1721 (2000). 11099404 10.1126/science.290.5497.1717 PMC2732363

[b36] Dunn J. *et al.* Pexophagy: the selective autophagy of peroxisomes. Autophagy 1, 75–83 (2005). 16874024 10.4161/auto.1.2.1737

[b37] Shintani T., Huang W. P., Stromhaug P. E. & Klionsky D. J. Mechanism of cargo selection in the cytoplasm to vacuole targeting pathway. Dev Cell 3, 825–837 (2002). 12479808 10.1016/s1534-5807(02)00373-8 PMC2737732

[b38] Hoeberichts F. A. & Woltering E. J. Multiple mediators of plant programmed cell death: interplay of conserved cell death mechanisms and plant-specific regulators. Bioessays 25, 47–57 (2002). 12508282 10.1002/bies.10175

[b39] Liang H. *et al.* Ceramides modulate programmed cell death in plants. Genes Dev. 17, 2636–2641 (2003). 14563678 10.1101/gad.1140503 PMC280613

[b40] Zhang L. Regulation of tomato responses to *Alternaria alternata* f. sp. *lycopersici* and AAL-toxin by plant hormones. *Doctor Thesis, Zhejiang University* (2011).

[b41] Jong A. J. d., Yakimova E. T., Kapchina V. M. & Woltering E. J. A critical role for ethylene in hydrogen peroxide release during programmed cell death in tomato suspension cells. Planta 214, 537–545 (2002). 11925037 10.1007/s004250100654

[b42] Liu L. *et al.* Ethylene independent induction of lycopene biosynthesis in tomato fruits by jasmonates. J. Exp. Bot. 63, 5751–5761 (2012). 22945939 10.1093/jxb/ers224 PMC3467294

[b43] Mase K. *et al.* Ethylene signaling pathway and MAPK cascades are required for AAL toxin-induced programmed cell death. Mol Plant Microbe Interact. 25, 1015–1025 (2012). 22512379 10.1094/MPMI-02-12-0036-R

[b44] Liang W. *et al.* The Arabidopsis homologs of CCR4-associated factor 1 show mRNA deadenylation activity and play a role in plant defence responses. Cell Res. 19, 307–316 (2009). 19065152 10.1038/cr.2008.317

[b45] Elmore J. M. & Coaker G. The role of the plasma membrane H^+^-ATPase in plant-microbe interactions. Mol Plant 4, 416–427 (2011). 21300757 10.1093/mp/ssq083 PMC3107590

[b46] Moore T., Martineau B., Bostock R., Lincoln J. & Gilchrist D. Molecular and genetic characterization of ethylene involvement in mycotoxin-induced plant cell death. Physiol Mol Plant Pathol. 54, 73–85 (1999).

[b47] Stefka, D. Spassievay, J. E. M. & Jacques Hille. The plant disease resistance gene *Asc-1* prevents disruption of sphingolipid metabolism during AAL-toxin-induced programmed cell death. Plant J. 32, 561–572 (2002). 12445127 10.1046/j.1365-313x.2002.01444.x

[b48] Filippou P., Antoniou C. & Fotopoulos V. Effect of drought and rewatering on the cellular status and antioxidant response of *Medicago truncatula* plants. Plant Signal Behav. 6, 270–277 (2011). 21330785 10.4161/psb.6.2.14633 PMC3121988

[b49] Zhou Y., Zhang Y., Zhao X., Yu H. & Shi K., Yu, Jing. Impact of light variation on development of photoprotection, antioxidants, and nutritional value in *Lactuca sativa L*. J. Agric. Food Chem. 57, 5494–5500 (2009). 19435354 10.1021/jf8040325

[b50] Hurkman W. J. & Tanaka C. K. Solubilization of plant membrane proteins for analysis by two-dimensional gel electrophoresis. Plant Physiol. 81, 802–806 (1986). 16664906 10.1104/pp.81.3.802 PMC1075430

[b51] Vizcaíno J. A. *et al.* ProteomeXchange provides globally coordinated proteomics data submission and dissemination. Nat Biotechnol 32, 223–226 (2014). 24727771 10.1038/nbt.2839 PMC3986813

[b52] Shilov I. V. *et al.* The Paragon Algorithm, a next generation search engine that uses sequence temperature values and feature probabilities to identify peptides from tandem mass spectra. Mol Cell Proteomics. 6, 1638–1655 (2007). 17533153 10.1074/mcp.T600050-MCP200

[b53] Eng J. K., McCormack A. L. & Yates J. R. An approach to correlate tandem mass spectral data of peptides with amino acid sequences in a protein database. J Am Soc Mass Spectrom. 5, 976–989 (1994). 24226387 10.1016/1044-0305(94)80016-2

[b54] Jiang X., Jiang X., Han G., Ye M. & Zou H. Optimization of filtering criterion for SEQUEST database searching to improve proteome coverage in shotgun proteomics. BMC bioinformatics 8, 323 (2007). 17761002 10.1186/1471-2105-8-323 PMC2040164

[b55] Lury D. A. & Fisher R. A. Statistical Methods For Research Workers, London. J R Stat Soc Ser C Appl Stat. 21 (1972).

[b56] Conesa A. *et al.* Blast2GO: a universal tool for annotation, visualization and analysis in functional genomics research. Bioinformatics 21, 3674–3676 (2005). 16081474 10.1093/bioinformatics/bti610

