# Supplementary material for: Critical Role of COI1-Dependent Jasmonate Pathway in AAL toxin induced PCD in Tomato Revealed by Comparative Proteomics
Source: Sci Rep. 2016 Jun 21;6:28451. doi: 10.1038/srep28451 (PMC4914994; doi:10.1038/srep28451)
Supplement: Supplementary Information [file srep28451-s1.doc]

## Supplementary information

### Critical Role of SlCOI1 Dependent Jasmonate Pathway in AAL toxin induced PCD Revealed by Comparative Proteomics

**Min Zhang^1, †^, Jin Koh^3, †^, Lihong Liu^1, 4, †^, Zhiyong Shao^1^, Haoran Liu^1^, Songshen Hu^1^, Ning Zhu^4^, Craig P. Dufresne^5^, Sixue Chen^3, 4^, Qiaomei Wang^1, 2*^**

^1^ Key Laboratory of Horticultural Plant Growth, Development and Quality Improvement, Ministry of Agriculture, Department of Horticulture, Zhejiang University, Hangzhou 310058, China

^2^ Proteomics and Mass Spectrometry, Interdisciplinary Center for Biotechnology Research, University of Florida, Gainesville, FL 32610, USA

^3^ Department of Biology, Genetics Institute, University of Florida, Gainesville, FL 32610, USA

^4^ Thermo Fisher Scientific, West Palm Beach, Florida 33407, USA

*^*^ Corresponding author*

*^†^These three authors contributed equally to this work.*

### Corresponding author

Qiaomei Wang

Professor

Tel: +86-571-85909333

Fax: 86-571-88766022

E-mail: [qiaomeiw@zju.edu.cn](mailto:qiaomeiw@zju.edu.cn)

## Supplementary Information Description

## Supplementary Figures

**Supplementary Figure S1**. Total protein number identified in two sets with 1% false discovery rate (FDR). Set 1 is colored in blue, set 2 is colored in green.

**Supplementary Figure S2.** Volcano plots of differential expression within four biological replicates of CA, CA+TA, CA+TA+JA, *jai1*+TA. Negative log10*p*-values were plotted on the y-axis, and log2 normalized fold change expression levels on the x-axis. Significant differential expression with *p*-value threshold values (α = 0.05) were detected at 0.8 and 1.2 of fold change threshold values. Red and green dots indicate up- and down-regulated protein, respectively.

**Supplementary Figure S3.** Comparison of functional classification between 10,367 overall detection and 2,670 differentially expressed proteins. Processes represented Blast2Go level 2-11 sorting. Each protein may be identified in more than one process.

**Supplementary Figure S4.** An outline of the sub-cellular localization classification of 10367 identified proteins. The bar chart shows the distribution of the non-redundant proteins into their sub-cellular localization. Processes represented Blast2Go level 2-11 sorting. Each protein may be identified in more than one organelle.

**Supplementary Figure S5.** Heat map of differentially expressed proteins in each comparison groups. The expression patterns of the differentially expressed proteins in each comparison groups were clustered and displayed based on the expression ratio as a log2 scale. The columns of the heat map represent samples and the rows represent proteins. Each row in the heat map indicates a single protein. The green and red colors indicate down- and up-regulation, respectively, in the WT, WT+JA+TA, *jai1*+TA relative to WT+TA.

## Supplementary Tables

**Supplementary Table S1-S9 are provided as separate excel sheets**

**Supplementary Table S1.** Complete list of the peptide information of proteins identified. A green color means down-regulation, while a red color shows up-regulation in protein level.

**Supplementary Table S2.** Complete list of the overall 10367 proteins identified. A green color means down-regulation, while a red color shows up-regulation in protein level.

**Supplementary Table S3.** List of 2670 differentially expressed proteins. A green color means down-regulation, while a red color shows up-regulation in protein level.

**Supplementary Table S4.** List of the 5715 phosphorylated proteins identified in tomato leaves. A green color means down-regulation, while a red color shows up-regulation in protein level.

**Supplementary Table S5.** List of the 4755 oxidized proteins identified in tomato leaves. A green color means down-regulation, while a red color shows up-regulation in protein level.

**Supplementary Table S6.** List of 472 differentially expressed proteins with phosphorylation. A green color means down-regulation, but a red color shows up-regulation.

**Supplementary Table S7.** List of 378 differentially expressed proteins with oxidation. A green color means down-regulation, but a red color shows up-regulation.

**Supplementary Table S8.** Comparative analysis of 267 proteins whose expression level were changed by phosphorylation. A green color means down-regulation, but a red color shows up-regulation.

**Supplementary Table S9.** Comparative analysis of 164 proteins whose expression level were changed by phosphorylation. A green color means down-regulation, but a red color shows up-regulation.

**Supplementary Table S10.** Comparison of expression patterns with phosphorylation across contrasts

**Supplementary Table S11.** Comparison of expression patterns with oxidation across contrasts

**Supplementary Table S12.** KEGG pathways with less than 40 sequences identified in the proteomics data.


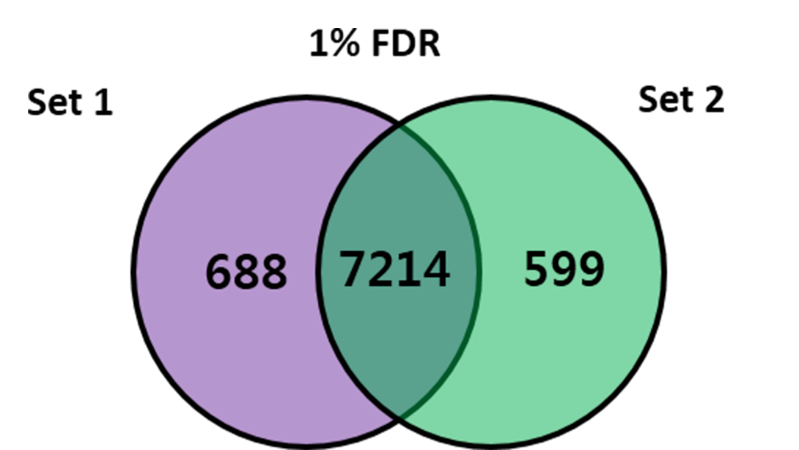


**Supplementary Figure S1**. Total protein number identified in two sets with 1% false discovery rate (FDR). Set 1 is colored in blue, set 2 is colored in green.

**
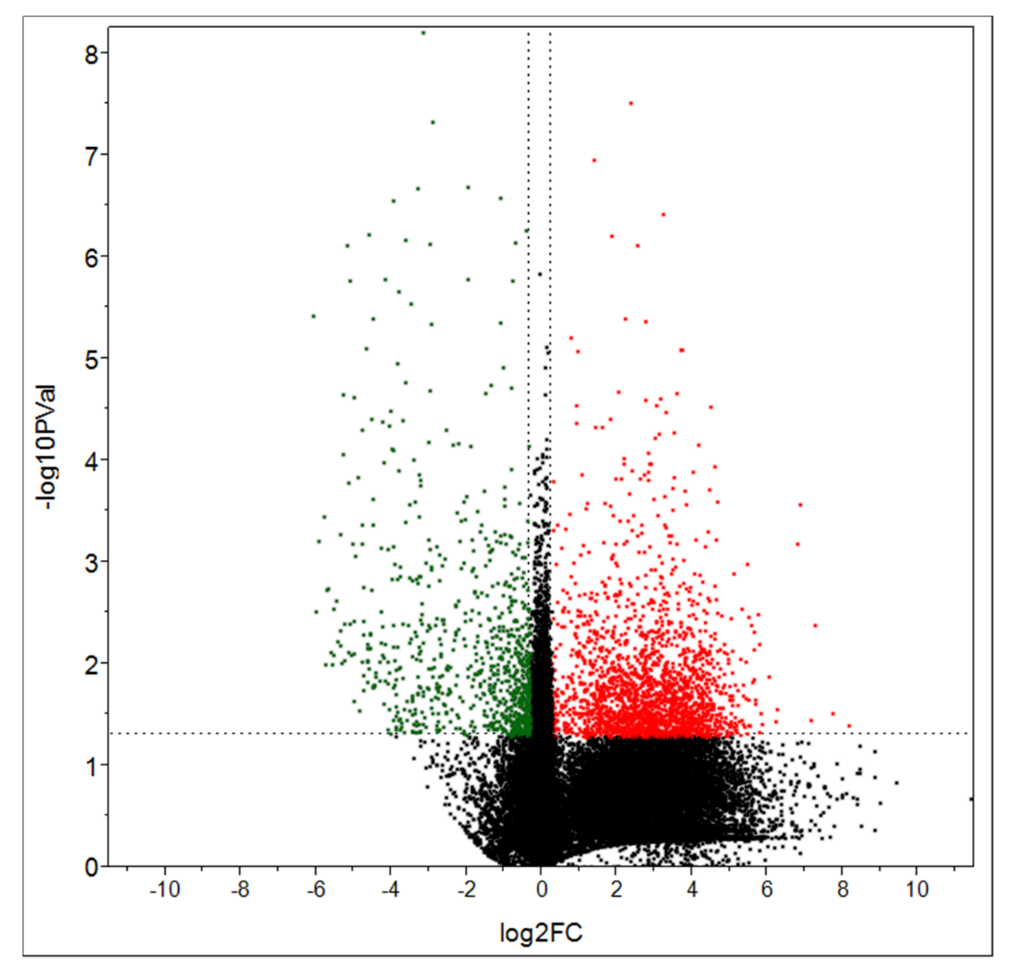
**

**Supplementary Figure S2.** Volcano plots of differential expression within four biological replicates of CA, CA+TA, CA+TA+JA, *jai1*+TA. Negative log10*p*-values were plotted on the y-axis, and log2 normalized fold change expression levels on the x-axis. Significant differential expression with *p*-value threshold values (α = 0.05) were detected at 0.8 and 1.2 of fold change threshold values. Red and green dots indicate up- and down-regulated proteins, respectively.

**
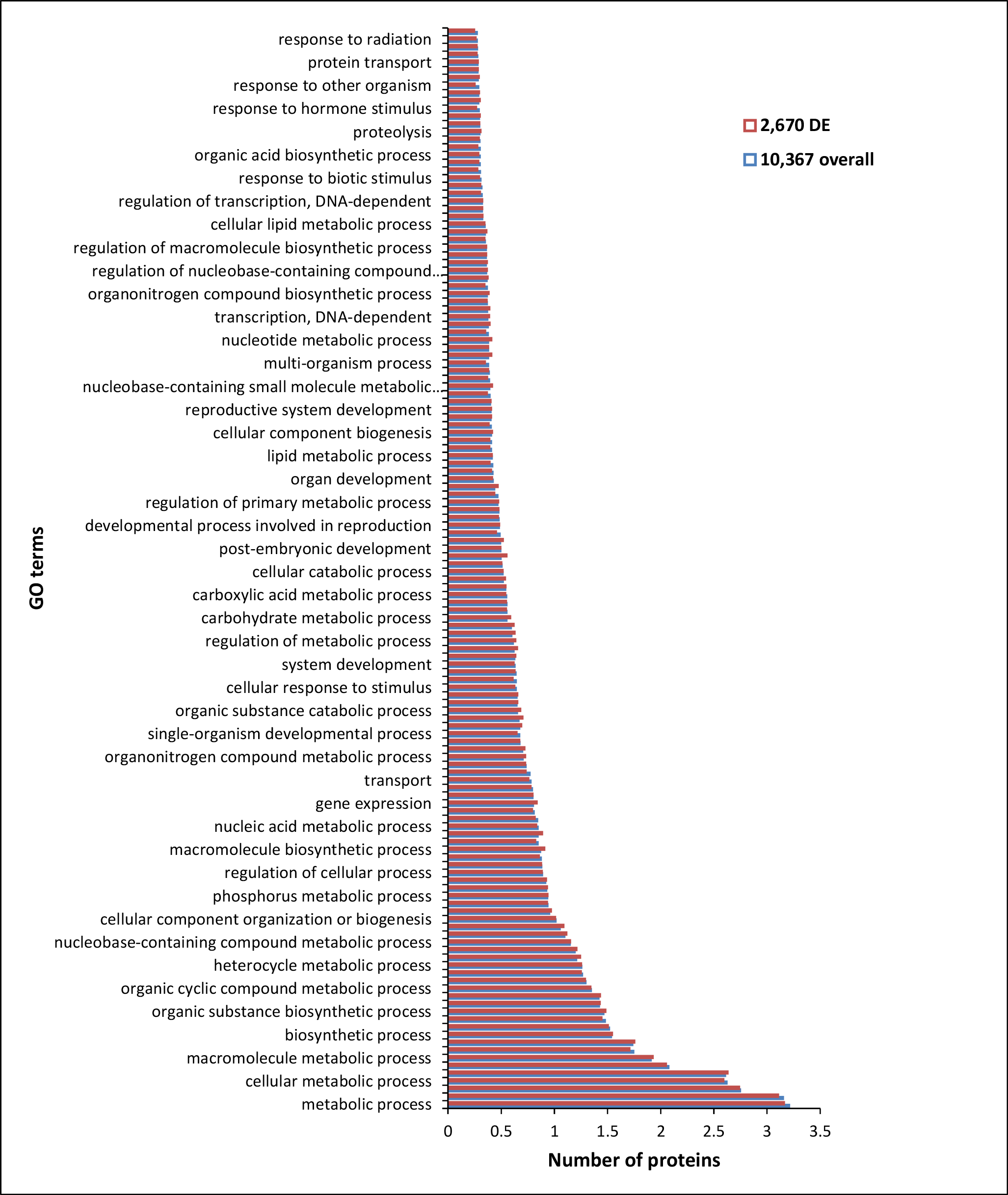
**

**Supplementary Figure S3.** Comparison of functional classification between 10,367 overall detection and 2,670 differentially expressed proteins. Processes represented Blast2Go level 2-11 sorting. Each protein may be identified in more than one process.

**
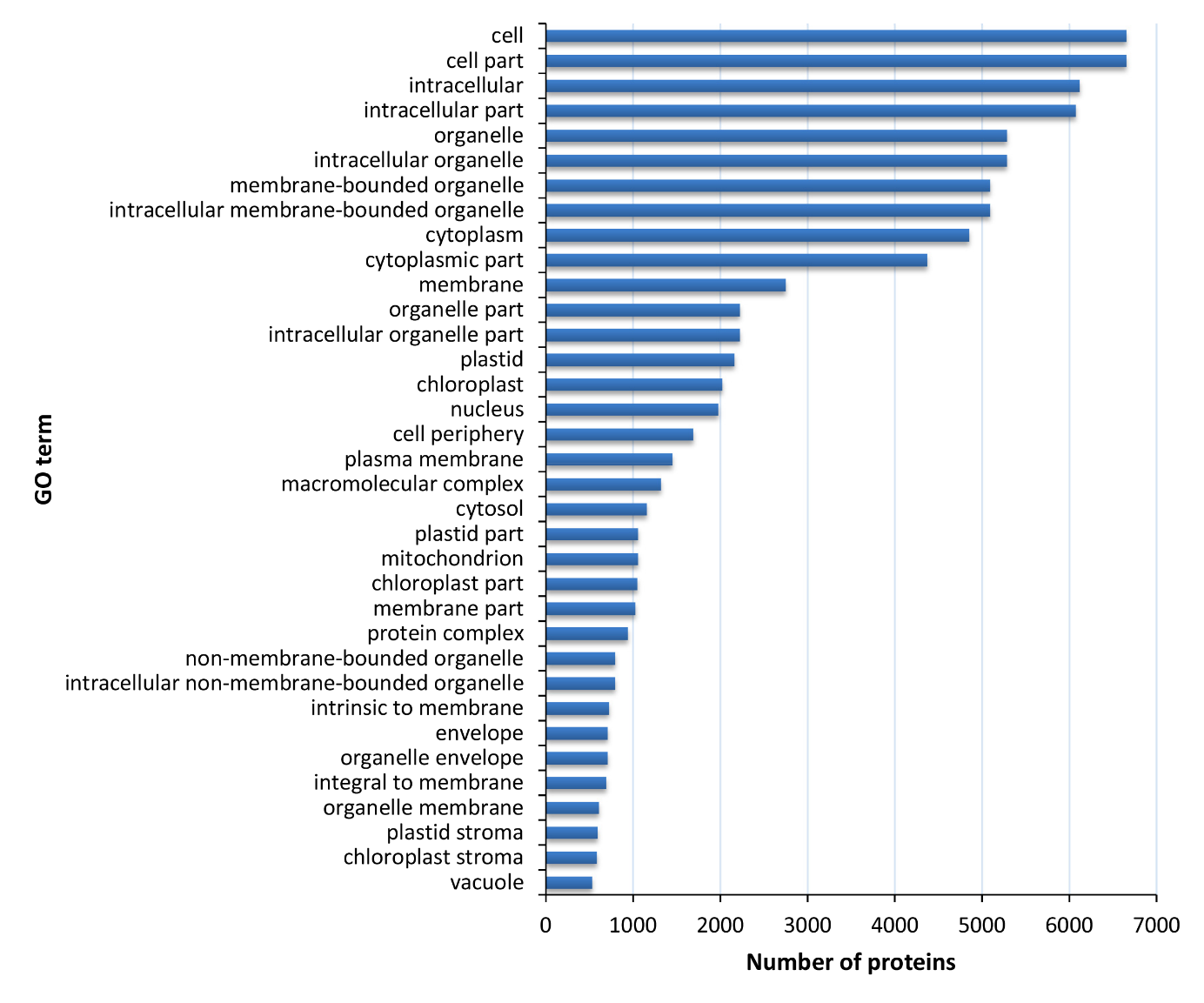
**

**Supplementary Figure S4.** An outline of the sub-cellular localization classification of 10367 identified proteins. The bar chart shows the distribution of the non-redundant proteins into their sub-cellular localization. Processes represented Blast2Go level 2-11 sorting. Each protein may be identified in more than one organelle.


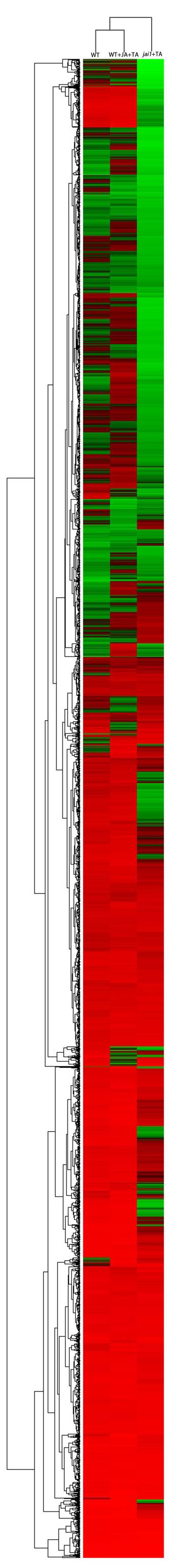


**Supplementary Figure S5.** Heat map of differentially expressed proteins in each comparison groups. The expression patterns of the differentially expressed proteins in each comparison groups were clustered and displayed based on the expression ratio as a log2 scale. The columns of the heat map represent genes and the rows represent samples. Each row in the color heat map indicates a single protein. The green and red colors indicate down- and up-regulation, respectively, in the WT, WT+JA+TA, *jai1*+TA relative to WT+TA.

**Supplementary Table S10. Comparison of expression patterns with phosphorylation across contrasts**

| **Type*^a^*** | **CA/(CA+TA)*^b^*** | **(CA+TA+JA) /(CA+TA)*^c^*** | **(*jai1*+TA) /(CA+TA)*^d^*** | **No. of**  **Protein** |
| --- | --- | --- | --- | --- |
| **I** | **↑*^e^*** | **-** | **-** | **37** |
| **II** | **↑** | **-** | **↑** | **8** |
| **III** | **↑** | **↑** | **-** | **4** |
| **IV** | **↑** | **↑** | **↑** | **1** |
| **V** | **-** | **-** | **↑** | **120** |
| **VI** | **-** | **-** | **↓** | **189** |
| **VII** | **-** | **↑** | **-** | **51** |
| **VIII** | **-** | **↑** | **↑** | **13** |
| **IX** | **-** | **↑** | **↓** | **6** |
| **X** | **-** | **↓** | **-** | **14** |
| **XI** | **-** | **↓** | **↑** | **1** |
| **XII** | **-** | **↓** | **↓** | **4** |
| **XIII** | **↓** | **-** | **-** | **13** |
| **XIV** | **↓** | **-** | **↑** | **4** |
| **XV** | **↓** | **-** | **↓** | **3** |
| **XVI** | **↓** | **↓** | **-** | **3** |
| **XVII** | **↓** | **↓** | **↑** | **1** |
| **XVIII** | **-** | **-** | **-** | **5,243** |
| **Total** |  |  |  | **5,715** |

*^a^* Types of changes of different comparisons.

*^b^* (WT/WT+TA) represented WT vs WT treated with TA.

*^c^* (WT+TA+JA/WT+TA) represented JA and TA treated WT vs TA treated WT.

*^d^* (*jai1*+TA/WT+TA) represented *jai1* treated with TA vs WT treated with TA.

*^e^* ↑, increased; ↓, decreased; –, not detected.

**Supplementary Table S11.** Comparison of expression patterns with oxidation across contrasts

| **Type*^a^*** | **CA/(CA+TA)*^b^*** | **(CA+TA+JA) /(CA+TA)*^c^*** | **(jai1+TA) /(CA+TA)*^d^*** | **No. of**  **Protein** |
| --- | --- | --- | --- | --- |
| **A** | **↑*^e^*** | **-** | **-** | **22** |
| **B** | **↑** | **-** | **↑** | **1** |
| **C** | **↑** | **-** | **↓** | **1** |
| **D** | **↑** | **↑** | **-** | **4** |
| **E** | **↑** | **↑** | **↑** | **1** |
| **F** | **↑** | **↑** | **↓** | **1** |
| **G** | **-** | **-** | **↑** | **93** |
| **H** | **-** | **-** | **↓** | **134** |
| **I** | **-** | **↑** | **-** | **48** |
| **J** | **-** | **↑** | **↑** | **9** |
| **K** | **-** | **↑** | **↓** | **4** |
| **L** | **-** | **↓** | **-** | **16** |
| **M** | **-** | **↓** | **↑** | **1** |
| **N** | **-** | **↓** | **↓** | **2** |
| **O** | **↓** | **-** | **-** | **24** |
| **P** | **↓** | **-** | **↑** | **6** |
| **Q** | **↓** | **-** | **↓** | **4** |
| **R** | **↓** | **↑** | **-** | **2** |
| **S** | **↓** | **↑** | **↑** | **1** |
| **T** | **↓** | **↓** | **-** | **3** |
| **U** | **-** | **-** | **-** | **4378** |
| **Total** |  |  |  | **4755** |

*^a^* Types of changes of different comparisons.

*^b^* (WT/WT+TA) represented WT vs WT treated with TA.

*^c^* (WT+TA+JA/WT+TA) represented JA and TA treated WT vs TA treated WT.

*^d^* (*jai1*+TA/WT+TA) represented *jai1* treated with TA vs WT treated with TA.

*^e^* ↑, increased; ↓, decreased; –, not detected.

**Supplementary Table S12.** KEGG pathways with less than 40 sequences identified in the proteomics data.

| **Classification*^a^*** | **KEGG pathways** | **Sequence number*^b^*** |
| --- | --- | --- |
| **Amino acid** | Histidine metabolism | 26 |
|  | Valine, leucine and isoleucine biosynthesis | 25 |
|  | Lysine biosynthesis | 23 |
|  | D-Alanine metabolism | 2 |
|  | D-Arginine and D-ornithine metabolism | 1 |
| **Lipid** | Linoleic acid metabolism | 38 |
|  | Steroid hormone biosynthesis | 30 |
|  | Sphingolipid metabolism | 29 |
|  | Terpenoid backbone biosynthesis | 28 |
|  | Fatty acid elongation | 21 |
|  | Glycosphingolipid biosynthesis - ganglio series | 15 |
|  | Steroid biosynthesis | 15 |
|  | Glycosphingolipid biosynthesis - globo series | 10 |
|  | Steroid degradation | 8 |
|  | Glycosphingolipid biosynthesis - lacto and neolacto series | 1 |
|  | Ether lipid metabolism | 7 |
| **Secondary metabolites** | Sulfur metabolism | 37 |
|  | Propanoate metabolism | 37 |
|  | Ubiquinone and other terpenoid-quinone biosynthesis | 36 |
|  | Tropane, piperidine and pyridine alkaloid biosynthesis | 34 |
|  | Butanoate metabolism | 34 |
|  | Nitrogen metabolism | 33 |
|  | Retinol metabolism | 32 |
|  | Arachidonic acid metabolism | 31 |
|  | Isoquinoline alkaloid biosynthesis | 30 |
|  | One carbon pool by folate | 24 |
|  | Nicotinate and nicotinamide metabolism | 24 |
|  | Flavone and flavonol biosynthesis | 24 |
|  | Riboflavin metabolism | 24 |
|  | Limonene and pinene degradation | 23 |
|  | Benzoate degradation | 22 |
|  | Streptomycin biosynthesis | 21 |
|  | Selenocompound metabolism | 21 |
|  | Geraniol degradation | 20 |
|  | Indole alkaloid biosynthesis | 20 |
|  | Anthocyanin biosynthesis | 20 |
|  | Other glycan degradation | 19 |
|  | Caffeine metabolism | 18 |
|  | Chloroalkane and chloroalkene degradation | 18 |
|  | Caprolactam degradation | 18 |
|  | C5-Branched dibasic acid metabolism | 16 |
|  | Vitamin B6 metabolism | 16 |
|  | Glycosaminoglycan degradation | 16 |
|  | Toluene degradation | 13 |
|  | Zeatin biosynthesis | 13 |
|  | Monoterpenoid biosynthesis | 12 |
|  | Novobiocin biosynthesis | 12 |
|  | Glycosaminoglycan biosynthesis - heparan sulfate / heparin | 12 |
|  | Various types of N-glycan biosynthesis | 11 |
|  | N-Glycan biosynthesis | 9 |
|  | Phenylpropanoid biosynthesis | 123 |
|  | Synthesis and degradation of ketone bodies | 9 |
|  | Chlorocyclohexane and chlorobenzene degradation | 9 |
|  | Styrene degradation | 9 |
|  | Carotenoid biosynthesis | 8 |
|  | Diterpenoid biosynthesis | 8 |
|  | Stilbenoid, diarylheptanoid and gingerol biosynthesis | 8 |
|  | Folate biosynthesis | 8 |
|  | Primary bile acid biosynthesis | 7 |
|  | D-Glutamine and D-glutamate metabolism | 7 |
|  | Fluorobenzoate degradation | 7 |
|  | Butirosin and neomycin biosynthesis | 6 |
|  | Sesquiterpenoid and triterpenoid biosynthesis | 6 |
|  | Isoflavonoid biosynthesis | 6 |
|  | Aflatoxin biosynthesis | 6 |
|  | Tetracycline biosynthesis | 6 |
|  | Glycosaminoglycan biosynthesis - chondroitin sulfate / dermatan sulfate | 6 |
|  | Naphthalene degradation | 5 |
|  | Taurine and hypotaurine metabolism | 4 |
|  | Polyketide sugar unit biosynthesis | 4 |
|  | Cutin, suberine and wax biosynthesis | 4 |
|  | Biosynthesis of ansamycins | 4 |
|  | Lipoic acid metabolism | 3 |
|  | Glucosinolate biosynthesis | 3 |
|  | Peptidoglycan biosynthesis | 3 |
|  | Ethylbenzene degradation | 3 |
|  | Biosynthesis of siderophore group nonribosomal peptides | 3 |
|  | Phosphonate and phosphinate metabolism | 3 |
|  | Xylene degradation | 1 |
|  | Mucin type O-Glycan biosynthesis | 1 |
|  | Brassinosteroid biosynthesis | 1 |
|  | Betalain biosynthesis | 1 |
|  | Penicillin and cephalosporin biosynthesis | 1 |
|  | Glycosylphosphatidylinositol(GPI)-anchor biosynthesis | 1 |
|  | Lipopolysaccharide biosynthesis | 1 |
|  | Glycosaminoglycan biosynthesis - keratan sulfate | 1 |

*^a^* KEGG pathways were classified by metabolic pathways of different molecules.

*^b^* Sequence number involved in the corresponding KEGG pathways above 40 were presented.
